# Identification of Rhododendron mariae extraction as a new attachment inhibitor against dengue virus by targeting the envelope protein domain III

**DOI:** 10.3389/fimmu.2025.1663878

**Published:** 2025-08-13

**Authors:** Chunyang Tian, Yuanru Zheng, Dongkai Tang, Haiyan Tian, Lingzhu Shi, Xuemei He, Jianhai Yu, Lijun Yan, Huihui Cao, Wei Zhao, Junshan Liu, Linzhong Yu, Zibin Lu

**Affiliations:** ^1^ Traditional Chinese Pharmacological Laboratory, Third Level Research Laboratory of State Administration of Traditional Chinese Medicine, School of Traditional Chinese Medicine, Southern Medical University, Guangzhou, China; ^2^ Guangdong Provincial Key Laboratory of Chinese Medicine Pharmaceutics, Southern Medical University, Guangzhou, China; ^3^ Guangdong Province Key Laboratory of Pharmacodynamic Constituents of TCM and New Drugs Research, College of Pharmacy, Jinan University, Guangzhou, China; ^4^ Guangdong Provincial Key Laboratory of Tropical Disease Research, School of Public Health, Southern Medical University, Guangzhou, China

**Keywords:** Rhododendron mariae extraction, dengue virus, antiviral, viral attachment, envelope protein domain III

## Abstract

**Introduction:**

Dengue virus (DENV), a mosquito-borne flavivirus, leads to over 390 million annual infections worldwide, and there are no approved antivirals so far. Rhododendron mariae (RM), a traditional Chinese herb abundant in flavonoids and triterpenoids, is used to treat respiratory disorders, yet its antiviral potential has been little explored. This study sought to assess the activity and mechanism of RM-1, an extract from RM, against DENV.

**Methods:**

In vitro, plaque reduction assays in BHK-21 cells were used to determine RM-1’s half-maximal effective concentration (EC50) against DENV-2. Its broad-spectrum activity against the four DENV serotypes was tested in Vero and Huh7 cells. In vivo, DENV-infected suckling mice were given RM-1 (10 mg/kg), with monitoring of viral loads, histology, and survival. Mechanistic studies included attachment assays and molecular docking to find potential targets.

**Results:**

RM-1 strongly inhibited DENV-2 (EC50=2.24 μg/mL) and showed dose-dependent activity against all four serotypes by blocking viral attachment. In infected mice, RM-1 lessened disease severity, reduced tissue lesions, lowered viral loads in serum, brain and spleen, and boosted survival rates. It targeted DENV envelope protein domain III (ED III), which is critical for host attachment.

**Discussion:**

This is the first report that RM-1 acts as a novel DENV attachment inhibitor by targeting ED III. These findings show RM-1’s promise as an anti-dengue therapeutic, supporting traditional herbs as sources of antivirals for flavivirus drug development.

## Graphical Abstract

The ethnopharmacological roots of *Rhododendron mariae* (used historically in Lingnan for respiratory ailments) inspired the development of *Rhododendron mariae* extraction (RM-1). RM-1 exhibits broad-spectrum anti-dengue activity against all four DENV serotypes (EC_50_: 1.62–2.79 μg/mL) by targeting the viral envelope domain III (ED III), blocking attachment (validated by SPR). *In vitro*, RM-1 suppresses viral progeny and cytopathic effects in BHK-21. It also reduces RNA and protein expression of DENV in BHK-21, Vero, Hepg2 and Huh7 cells. *In vivo*, it reduces viral loads, mitigates tissue damage, and improves survival in DENV-infected suckling mice. This study bridges traditional herbal knowledge with modern drug discovery, positioning RM-1 as the first ED III-targeted therapeutic candidate against dengue.

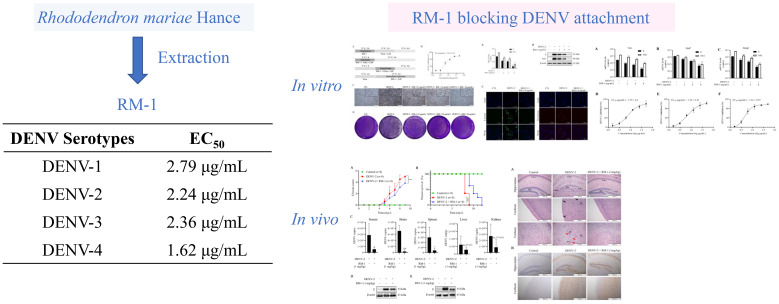

## 1 Introduction

Dengue virus (DENV) is a single-stranded RNA virus classified within the Flavivirus genus of the Flaviviridae family. It is primarily transmitted to humans through the bites of infected *Aedes aegypti* mosquitoes, making it the most widespread arthropod-borne viral infection in tropical and subtropical regions globally (1). DENV exists in four distinct serotypes (DENV-1 to DENV-4), each capable of inducing infection. While primary infections often lead to mild or moderate illness, secondary infections with heterologous serotypes significantly increase the risk of severe complications, such as dengue hemorrhagic fever or dengue shock syndrome (2). Upon entering host cells, the genomic RNA of DENV is translated into three structural proteins—prM (pre-membrane), E (envelope), and C (capsid)—along with seven non-structural (NS) proteins: NS1, NS2A, NS2B, NS3, NS4A, NS4B, and NS5. These proteins work together to support viral replication, assembly, and immune evasion within the host (3). Clinically, DENV infection presents with a broad spectrum of symptoms, ranging from mild dengue fever (DF), which includes high fever, retro-orbital pain, and rashes, to more severe forms, characterized by plasma leakage, hemorrhage, and multi-organ failure (4). Epidemiological studies estimate that nearly 390 million individuals contract DENV annually, resulting in approximately 25,000 deaths across over 100 nations (5). Despite its significant global health impact, no specific antiviral treatment or universally approved vaccine exists, leaving current disease management strategies heavily reliant on vector control and supportive care (6). This pressing situation highlights the urgent necessity for developing effective antiviral therapies and preventive measures to combat dengue infections and reduce disease burden globally.

Rhododendron mariae (RM), also known as Lingnan Rhododendron, refers to the dried leaves or shoots of *Rhododendron mariae* Hance, which is widely distributed throughout the Lingnan region, particularly in Guangdong province. As a popular traditional Chinese medicinal herb, it is currently used in the clinical management of chronic bronchitis and asthm (7). Numerous phytochemical investigations of RM have revealed the presence of flavonoids, triterpenoids, phenols, organic acids, etc. However, the modern pharmacological studies so far are mainly focused on its antitussive and expectorant activities, thereby substantially limiting the development of clinical applications of RM (8).

In this study, we firstly reported that a fraction from RM (RM-1) exerts remarkably antiviral effects against DENV infection both *in vitro* and *in vivo*. These results suggest that RM-1 holds promise as a potential therapeutic for dengue infection and provide a promising clinical application of RM-1 to treat DENV.

## 2 Materials and methods

### 2.1 Materials

RM was provided by Guangzhou Baiyun Mountain Hutchison Whampoa Traditional Chinese Medicine Co., Ltd. (Guangzhou, Guangdong, China) and authenticated by researcher Huagu Ye (South China Botanical Garden, Guangzhou, China). The baby hamster kidney cell line BHK-21, mosquito larva C6/36 cell, three serotypes of DENV (DENV-1, DENV-3 and DENV-4) and the purified ED III protein were kindly gifts from Professor Wei Zhao (Southern Medical University). Human hepatocellular carcinoma cell lines Huh7 and HepG2 were purchased from the Institute of Biochemistry and Cell Biology of the Chinese Academy of Sciences (Shanghai, China). DENV-2 New Guinea C derivative strain was kindly provided by Professor Xingang Yao (Southern Medical University). The virus was propagated in C6/36 cells and stored at −80°C until further use. The main reagents and antibodies are in supplementary material.

### 2.2 Extraction and purification

The extraction was performed following previously established protocols (9). The dried and coarsely powdered raw material (RM, 3.5 kg) was extracted with 70% ethanol in an ultrasonic bath (1.5 h per extraction, repeated three times). The combined extracts were concentrated under reduced pressure to afford a residue (122.9 g). Subsequently, the residue was subjected to purification using a D101 macroporous resin column with an eluent of ethanol-water (80:20, *v*/*v*). Further purification was performed by preparative high-performance liquid chromatography (HPLC) using a mobile phase of methanol/water (70:30, *v*/*v*) under the following conditions: flow rate = 0.4 mL/min; detection wavelength = 254 nm; column temperature = 30°C. This process yielded the target fraction (RM-1).

The freeze-dried RM-1 powder was dissolved in acetonitrile (10 μg/mL) and centrifuged at 12,000 ×g for 10 minutes. The resulting supernatant was filtered through a 0.22 μm microporous membrane before performing LC-MS analysis. Chromatographic separation was conducted using an Agilent SB-C18 column (100 mm × 3.0 mm, 1.8 μm; Agilent Technologies, California, USA) maintained at 30°C. The mobile phases consisted of water containing 0.1% acetic acid (A) and acetonitrile (B), with an optimized gradient elution program (0–27 min, 5–50% B). The flow rate was set to 0.4 mL/min, and the injection volume was 2 μL. Mass spectrometry (MS) analysis was performed using ultra-high-performance liquid chromatography (UHPLC) coupled with Orbitrap high-resolution mass spectrometry (HRMS; Thermo Fisher Scientific, MA, USA) equipped with an electrospray ionization (ESI) source. Data acquisition was conducted in both positive and negative ionization modes over a mass range of m/z 100–1000. The MS parameters included: a full MS resolution of 60,000, MS/MS resolution of 15,000, capillary voltage of 3.5 kV (ESI+) or −2.5 kV (ESI−), ion transfer tube temperature of 320°C, vaporizer temperature of 350°C, nitrogen as the sheath gas (40 Arb) and auxiliary gas (10 Arb), and higher-energy collisional dissociation (HCD) fragmentation mode with 30% collision energy.

### 2.3 Cell culture

BHK-21 and Huh7 cells were maintained in RPMI-1640 medium (Gibco, Netherlands) supplemented with 10% fetal bovine serum (FBS, ExCell Bio, Shanghai, China) and, cultured at 37°C in a 5% CO_2_ incubator. In contrast, C6/36 cells were grown in RPMI-1640 medium containing 10% FBS (Gibco, Netherlands), 100 U/mL penicillin, and 100 μg/mL streptomycin (Gibco, Netherlands). These cells were incubated at 28°C.

### 2.4 Cell viability assay

BHK-21 cells were grown into 96-well plates at a density of 0.8 × 10^4^ cells per well and allowed to adhere and proliferate for 24 hours at 37°C. Following incubation, the medium was replaced with RPMI-1640 supplemented with 2% FBS, and the cells were treated with increasing concentrations of RM-1. The treatment was maintained for a period of 4 days. Subsequently, 100 μL of MTT solution (0.5 mg/mL) was added to each well of the 96-well plate, and the cells were incubated for an additional 4 hours at 37°C. During this incubation, viable cells metabolized the MTT reagent, forming purple formazan crystals. After the incubation, the formed formazan was solubilized by adding 100 μL of dimethyl sulfoxide (DMSO) to each well. The absorbance of the resulting solution was then measured at 490 nm using a microplate reader (Thermo Fisher Scientific, Waltham, USA).

### 2.5 Prophylactic activity

BHK-21 cells were grown into 96-well plates at a density of 0.8 × 10^4^ cells per well and incubated at 37°C for 24 hours. Different concentrations of RM-1 (0.625 μg/mL ~ 20 μg/mL) were added 5 hours before infection with 200 PFU of DENV-2. After an hour infection period, the cells were washed twice with PBS (pH 7.2) to remove unbound virus particles. The cells were then cultured in RPMI-1640 medium supplemented with 2% FBS at 37°C for 4 additional days. The prophylactic effects of RM-1 against DENV-2 were evaluated using the CCK-8 assay (10).

### 2.6 Anti-attachment activity

BHK-21 cells were exposed to 200 PFU of DENV-2 in the presence or absence of RM-1 (0.625 μg/mL ~ 20 μg/mL) at 4°C for 1 h. Next, the infected cells were washed by sterile PBS twice to remove unabsorbed viruses and further cultured at 37°C for another 4 d in the presence of 5% CO_2_. The antiviral effects of RM-1 on the adsorption phase of DENV-2 were evaluated using the CCK-8 assay (11).

### 2.7 Anti-entry activity

BHK-21 cells were exposed to 200 PFU of DENV-2 and incubated at 4°C for 1 hour to facilitate virus attachment to the cell surface. After two washes with PBS, the cells were incubated at 4°C for an additional 1 hour with or without RM-1 (0.625 μg/mL ~ 20 μg/mL). Following incubation, the supernatants were discarded, and the cells were cultured in RPMI-1640 medium supplemented with 2% FBS at 37°C for 4 days. The antiviral effects of RM-1 on DENV-2 entry were evaluated using the CCK-8 assay (11).

### 2.8 Anti-intracellular replication activity

BHK-21 cells were exposed to 200 PFU of DENV-2 and incubated at 37°C for 1 hour. Afterward, the cells were washed twice with PBS and cultured at 37°C with or without RM-1 (0.625 μg/mL ~ 20 μg/mL). The antiviral effect of RM-1 on the intracellular replication of DENV-2 was evaluated after 4 days of incubation using the CCK-8 assay.

### 2.9 CCK-8 assay

The assay was performed using the CCK-8 kit (Keygen Biotech, Jiangsu, China), and the procedure was followed as outlined by the manufacturer. Briefly, cells were incubated with the CCK-8 solution, and after an hour the absorbance was measured at 490 nm using a microplate reader. This method allowed for the accurate quantification of cell proliferation and cytotoxicity induced by various treatments, such as antiviral compounds.

### 2.10 Cytopathic effect

BHK-21 cells were initially seeded into a 6-well plate at a density of 1 × 10^5^ cells per well and incubated at 37°C for 24 hours. Afterward, the cells were exposed to 200 PFU of DENV-2 and incubated at 4°C for 1 hour, either with or without RM-1 at concentrations (2 μg/mL, 4 μg/mL, and 8 μg/mL). Following infection, the cells were washed twice with PBS and cultured in RPMI-1640 medium supplemented with 2% FBS at 37°C for 4 additional days. The cytopathic effects, resulting from DENV-2 infection, were observed and documented using an IX 53 light microscope (Olympus, Tokyo, Japan).

### 2.11 Plaque assay

To assess the antiviral effects of RM-1, BHK-21 cells were treated with varying concentrations (2 μg/mL, 4 μg/mL, and 8 μg/mL), alongside infection with DENV-2. Following a 2-day incubation period after infection (d.p.i.), the supernatants containing progeny virus were collected and used to infect fresh BHK-21 cells. The cells were then incubated at 37°C for 1 h. After attachment, the medium was replaced with RPMI-1640 containing 2% FBS and 1.2% methyl cellulose to facilitate plaque formation. The infected cells were incubated for 5 to 6 additional days to allow plaque development, as DENV plaques develop more slowly. After the incubation period, plaques were fixed with 4% PFA and stained for 15 minutes with 2% crystal violet solution. Plaque formation was subsequently assessed and quantified to determine the impact of RM-1 treatment on viral replication, as detailed in prior research (12).

### 2.12 Quantitative real-time PCR

RNA extraction from intracellular samples was carried out using RNAiso Plus (Takara, Shiga, Japan), following the manufacturer’s protocol to ensure high-quality RNA. Complementary DNA (cDNA) for subsequent analysis was generated using PrimeScript™ RT Master Mix (Takara, Shiga, Japan) for reverse transcription. Quantitative reverse transcription polymerase chain reaction (qRT-PCR) was then conducted using a LightCycler^®^ 96 real-time PCR system (Roche, Switzerland) with Bestar R qPCR Master Mix (DBI Bioscience, Shanghai, China). The reaction conditions: 20 μL reaction volume containing 10 μL 2× TB Green Premix, 0.4 μL of each primer (final concentration: 0.2 μM), 0.2 μL of probe (final concentration: 0.1 μM), 2 μL of 5-fold diluted cDNA template, and nuclease-free water to volume. Thermal cycling conditions were: 95°C for 30 s, followed by 40 cycles of 95°C for 5 s and 60°C for 34 s (annealing/extension). Fluorescence was detected at the end of each 60°C step. The primers and TaqMan probes for detecting DENV-2-specific genes were optimized for the E and NS1 genes. The sequences of the primers and probes are detailed as follows: for the E gene, the forward primer was 5′-CAGTCGGAAATGACACAG-3′, the reverse primer was 5′-GCAACACCATCTCATTGA-3′, and the probe was 5′-FAM-AAGTAACACCACAGAGTTCCATCACA-BHQ1-3′. For the NS1 gene, the forward primer was 5′-CTTGAGATGGACTTTGATTTCTGC-3′, the reverse primer was 5′-CTCTTCTTTCTCTTTCAATGGTCTG-3′, and the probe was 5′-FAM-ACTCATAACAGAATGGTGCTGCCGATC-BHQ1-3′. For β-actin gene, the forward primer was 5′-CCGTTGCCCTGAGGCTCTTT-3′, and the reverse primer was 5′-GGTCTTTGCGGATGTCCACG-3′. To quantify mRNA expression levels accurately, standard curves were generated using plasmid cDNA of the E gene (pMD18-T-DENV-2-E) and the NS1 gene (pMD18-T-DENV-2-NS1), enabling precise quantification of DENV-2 gene expression in the samples.

### 2.13 Western blot analysis

Proteins from the samples were extracted using a carefully prepared lysis buffer, containing key reagents such as Tris, Triton X-100, and sodium chloride, among others. This buffer composition facilitates effective protein solubilization while preserving protein integrity. The extraction was carried out under cold conditions (4°C) to minimize protein degradation. After centrifugation, the supernatant was retained for analysis and stored at −20°C to prevent protein degradation. The total protein concentration was determined using a BCA assay kit, which provides a reliable method for quantification.

For Western blotting, equal amounts of total protein were separated using SDS-PAGE. The proteins were then transferred to PVDF membranes, a process that allows for efficient protein immobilization. To prevent nonspecific binding, the membranes were blocked with a 5% (w/v) skim milk solution in TBS-T for 1 hour. Primary antibodies specific for the E protein, NS1 protein, and β-actin were used for the detection of DENV-specific proteins and loading control, respectively. Following incubation with primary antibodies at 4°C overnight, the membranes were washed to eliminate any unbound or non-specifically bound antibodies. Secondary antibodies conjugated with a detection enzyme were then applied to facilitate the visualization of the protein bands. Following a second incubation at 4°C, the membranes were washed again, and protein bands were detected using the FluorChem ETM system, with signal enhancement through an ECL kit.

### 2.14 Immunofluorescence assay

At 48 hours after infection, the cells were carefully prepared for immunofluorescence imaging to evaluate the expression and subcellular localization of viral proteins. Initially, the cells were gently rinsed with pre-warmed PBS to eliminate any residual medium and cellular debris, ensuring clear visibility under the microscope. Fixation was performed using 4% paraformaldehyde, which preserves cellular structures and immobilizes proteins, followed by permeabilization with 0.2% Triton X-100 to enable antibody access to intracellular antigens. Blocking with 5% skim milk prevented nonspecific antibody binding, ensuring precise detection of target proteins. The primary antibodies targeting the viral E and NS1 proteins were diluted to final concentrations of 1:500 and 1:400, respectively, and incubated with the cells overnight at 4°C. This prolonged incubation allows optimal binding of antibodies to their respective antigens. After primary antibody binding, cells were washed. Subsequently, they were incubated with Alexa Fluor 488-conjugated secondary antibodies, which fluoresce under specific wavelengths, for 2 hours at 4°C. Nuclear staining with DAPI added contrast to the images by labeling DNA. Finally, the stained cells were washed and mounted for visualization. The images were captured using a confocal microscope (LSM800, Carl Zeiss, Oberkochen, Germany) to assess the expression and localization of the viral proteins (13).

### 2.15 DENV-infected mouse model

ICR mice, procured from SPF (Beijing) Biotechnology Co., Ltd, were housed in a pathogen-free environment under biosafety level-2 conditions at the Animal Experimental Center of Guangzhou University of Chinese Medicine. All animal experiments were conducted in accordance with ethical guidelines, and ethical approval was obtained from the university’s Ethics Committee (Permit number: 20201217003). Seven-day-old suckling mice were selected due to their susceptibility to DENV-2 infection, facilitating the evaluation of antiviral efficacy. The mice were administered DENV-2 intracerebrally at a dose of 400 PFU and intraperitoneally at 4×10^5^ PFU to establish systemic infection. Control mice received RPMI-1640 medium instead of the virus.

To evaluate the therapeutic potential of RM-1, mice were treated intraperitoneally with 1 mg/kg of the compound on alternate days (1-, 3-, 5-, and 7-days post-infection), while control groups were given an equivalent volume of vehicle. The clinical progression of DENV-2 infection was assessed daily using a standardized scoring system that categorized illness into six grades: 0 indicating a healthy state; 1 representing mild symptoms (such as decreased mobility and hunched posture); 2 corresponding to limbic seizures; 3 denoting dyspraxia (with weakness in either forelimbs or hindlimbs); 4 signifying paralysis; and 5 indicating death. This approach allowed a detailed analysis of disease severity, including mobility issues, seizures, and paralysis.

On the sixth day post-inoculation, the mice were euthanized, and tissues, including blood, brain, liver, kidneys, and spleen, were harvested for molecular and histological assessments. These samples were used for qRT-PCR to quantify viral RNA, Western blotting to detect protein expression, histopathology to examine tissue damage, and immunohistochemistry to localize viral antigens. Survival rates were monitored daily across a 12-day observation period to evaluate the compound’s overall protective effect.

### 2.16 Histology and immunohistochemistry

Histological examination of tissue samples was carried out on paraffin-embedded sections fixed in paraformaldehyde. These samples were sliced into 4 μm-thick sections and stained with hematoxylin and eosin (H&E) to visualize cellular structures and detect pathological changes. The sections were evaluated using an Olympus IX 53 light microscope to document alterations such as cellular infiltration, tissue damage, and necrosis. For immunohistochemistry, the paraffin sections were initially dewaxed in xylene and rehydrated through a descending ethanol gradient. the sections were subjected to antigen retrieval by heating in citrate buffer (pH 6.0) at boiling temperature for 10 minutes. Endogenous peroxidase activity, which could interfere with staining, was blocked by incubating in 3% hydrogen peroxide. After blocking nonspecific antibody binding sites with 5% bovine serum albumin (BSA), the sections were incubated overnight at 4°C with a primary antibody. The samples were then treated with a secondary antibody using the GTVision™ III Detection System (Dako, Denmark), conjugated to horseradish peroxidase (HRP). After 30 minutes, color development was performed with 3,3′-diaminobenzidine (DAB) at room temperature, which forms a brown precipitate at the site of antibody binding, and the reaction was terminated after 5 minutes to prevent overstaining. The stained sections were rinsed thoroughly and visualized under an Olympus IX 53 light microscope.

### 2.17 Statistical analysis

Data were presented as the mean ± standard deviation (SD). The half-maximal cytotoxic concentration (CC50) and the half-maximal effective concentration (EC50) were determined using GraphPad Prism 8.0 software (San Diego, CA, USA). Statistical analysis was conducted using one-way analysis of variance (ANOVA). Differences with a P-value less than 0.05 were considered statistically significant.

## 3 Results

### 3.1 RM-1 inhibits the attachment of DENV-2 to the cell surface during infection

**Figure 1:**
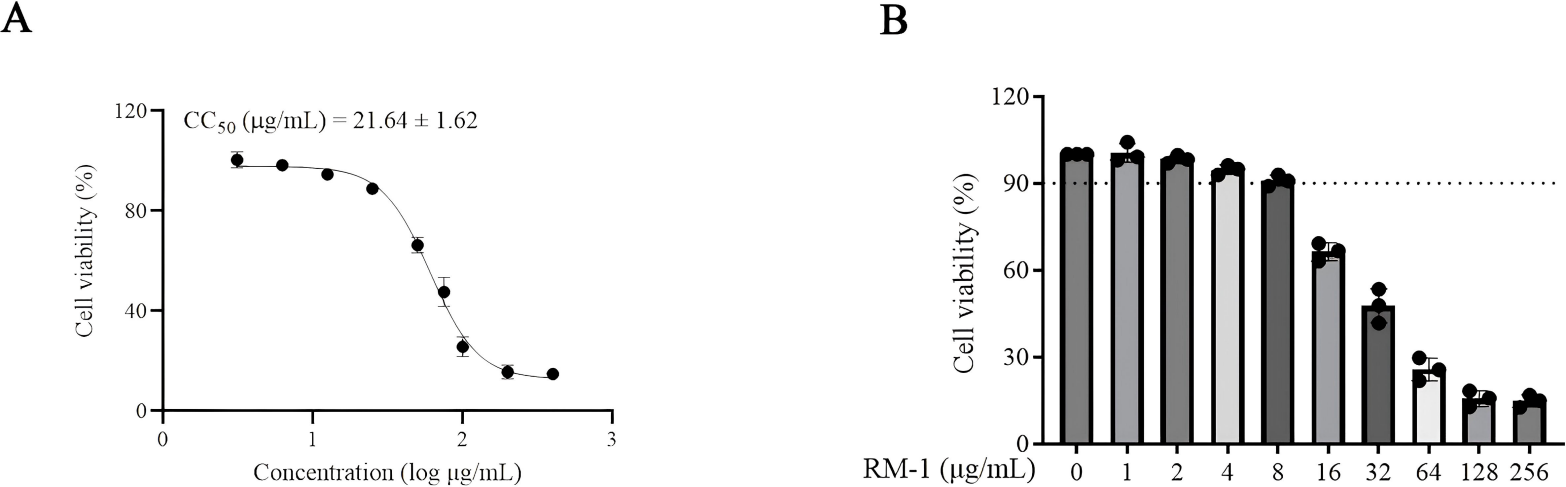
The molecular structure and cytotoxicity of RM-1. **(A, B)** cytotoxicity and CC_50_ of RM-1 on BHK-21 cells measured by MTT assay. Data were expressed as mean ± SD of three independent experiments.

**Figure 2:**
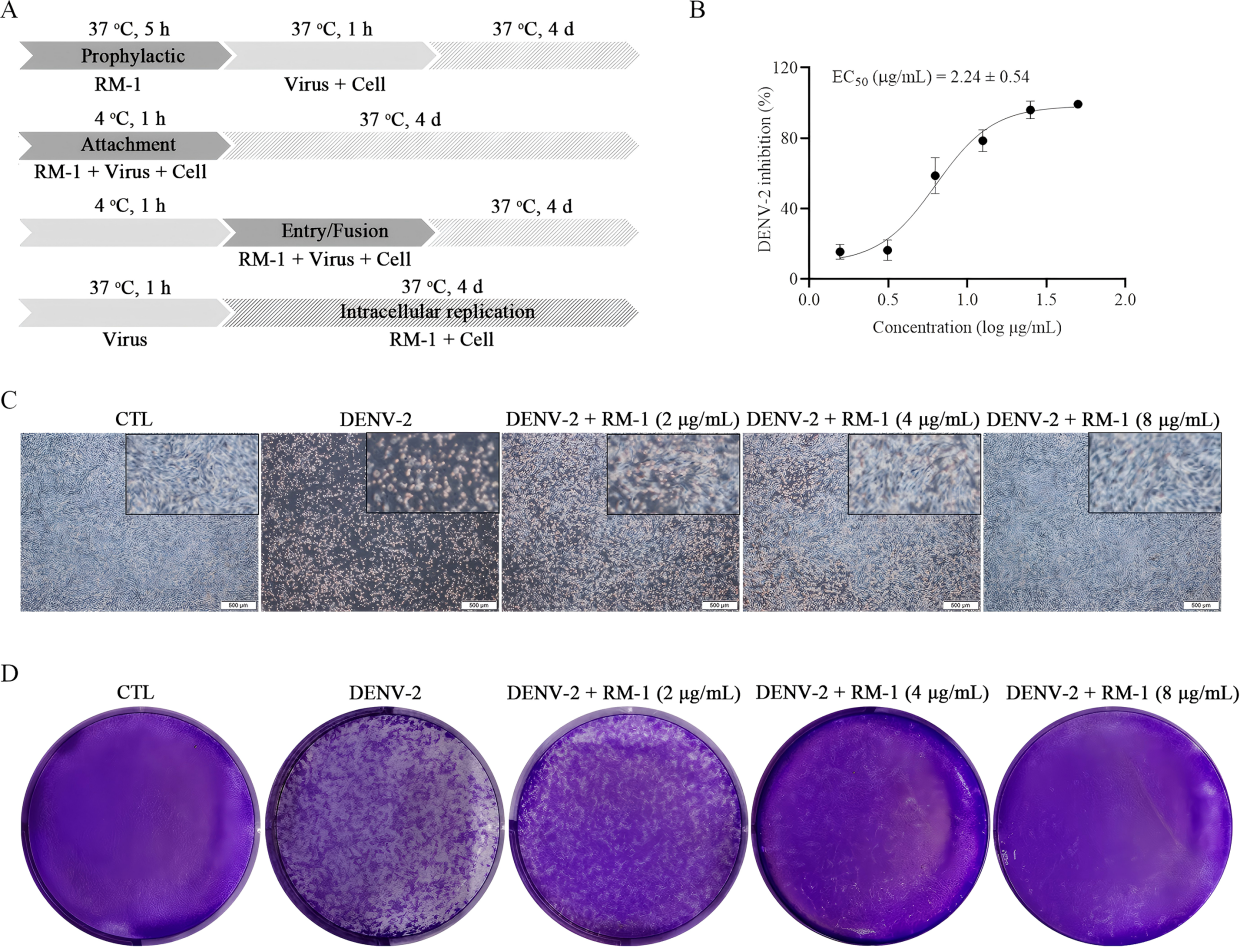
Effects of RM-1 on the attachment process in DENV-2 infection. **(A)** An operating schematic diagram of RM-1 on different stages of viral infection. **(B)** EC_50_ of RM-1 on viral attachment. **(C)** Cytopathic effects of RM-1 on DENV-2-infected BHK-21 cells. **(D)** Virus plaques on progeny DENV-2-infected BHK-21 cells. Data were expressed as mean ± SD of three independent experiments.

MTT assay was firstly conducted to assess the cytotoxic effects of RM-1 on BHK-21 cell line. The CC_50_ value of RM-1 was found to be 21.62 μg/mL (shown in **Figures 1A, B**). Next, a time-of-addition assay was conducted to determine whether RM-1 exerts its antiviral effects at a specific stage of the viral life cycle in vitro. Coinciding with infection time, RM-1 was added during various stages of infection: prophylactic, viral attachment, viral entry/fusion and intracellular replication (shown in **Figure 2A**). The results of CCK-8 assays indicated that RM-1 exerted antivirus activities only on the viral attachment process with an EC_50_ of 2.24 μg/mL (shown in **Figure 2B**), while no obvious effects were observed in the other three stages (shown in **Supplementary Figures S1A–C**). Therefore, the concentrations closed to EC_35_, EC_65_ and EC_95_ values (2 μg/mL, 4 μg/mL and 8 μg/mL) were selected for the following viral attachment tests in vitro.

CPE, the cell degeneration caused by virus infection, is a crucial reference standard for the assessment of antiviral effects (13). To further evaluate the antiviral effects of RM-1, CPEs on BHK-21 cells were and documented using a microscope. Compared with DENV-infected cells, RM-1 significantly reduced the cytopathic change in a dose-dependent manner, especially at a concentration of 8 μg/mL. (shown in **Figure 2C**). Collectively, these results show that RM-1 had anti-DENV effects by blocking DENV-2 attachment to the cell surface in its whole life cycle.

### 3.2 RM-1 reduces the synthesis of progeny DENV-2

The determination of progeny virus is commonly used to assess the antiviral efficacy of a treatment (14). To measure the production of progeny virus, the DENV-2 titers in supernatants of infected BHK-21 cells, in the presence or absence of RM-1, were quantified using a plaque assay. The results indicated that viral plaque formation was obviously inhibited by RM-1 (shown in **Figure 2D**), implying the lower synthesis of progeny DENV-2 after the treatment of RM-1.

### 3.3 RM-1 reduces the RNA and protein expression levels of DENV-2

**Figure 3:**
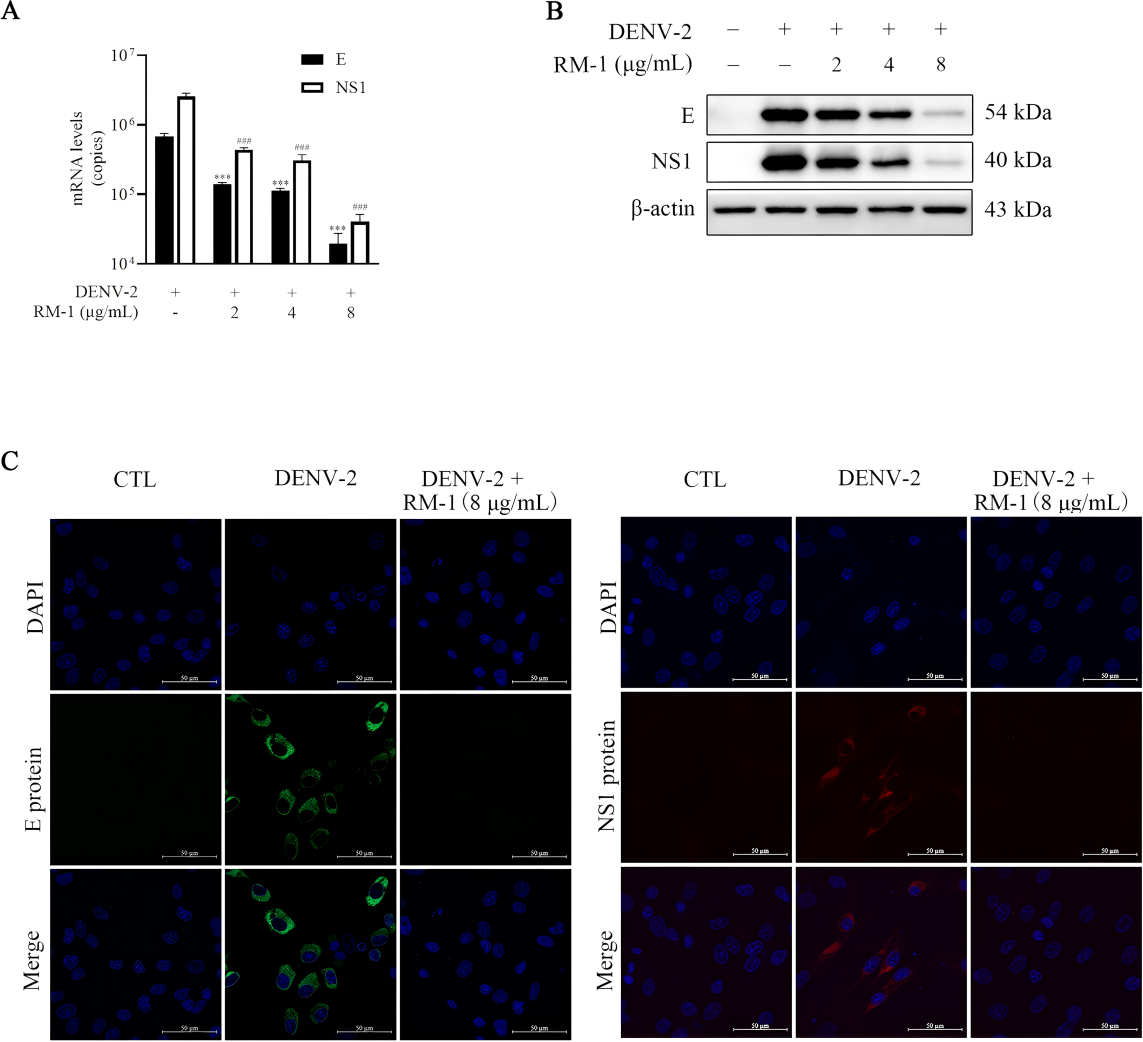
Effects of RM-1 on DENV-2 RNA and protein levels. BHK-21 cells were infected with DENV-2 containing RM-1. After 1 h of infection at 4°C, cells were washed and added with maintenance media. **(A)** The RNA levels of E and NS1 detected by qRT-PCR at 48 h.p.i. **(B)** The protein levels of E and NS1 proteins detected by Western blot analysis at 48 h.p.i. **(C)** Immunofluorescence levels of E and NS1 protein at 48 h.p.i. ^###^ *P* < 0.001 *versus* DENV group in NS1 RNA, ^***^ *P* < 0.001 *versus* DENV group in the RNA level of E protein RNA by one-way ANOVA with Tukey’s test.

Structure and non-structure proteins are indispensable for the entire viral life circles (15). The E protein, one of the three structural proteins, facilitates viral attachment to the cell surface by binding to specific receptors (16). The NS1 protein, a highly conserved non-structural component, is essential for the synthesis of DENV progeny (17). To assess RNA levels of the E and NS1 proteins, a qRT-PCR assay was conducted. As anticipated, the RNA copy numbers for both E and NS1 proteins were notably reduced by more than 97% in the presence of RM-1 (8 μg/mL) (shown in **Figure 3A**). Furthermore, Western blot analysis confirmed that RM-1 caused a dose-dependent reduction in the expression levels of both E and NS1 proteins (shown in **Figure 3B**). In addition, consistent with our results above, green (E protein) or red (NS1 protein) fluorescence signals were almost completely disappeared by RM-1 (8 μg/mL) treatment (shown in **Figure 3C**). These data further confirm a significant down-regulation of E and NS1 protein levels in DENV-infected BHK-21 cells treated with RM-1.

### 3.4 RM-1 inhibits infectivity of DENV in different cell lines and in all four DENV serotypes

**Figure 4:**
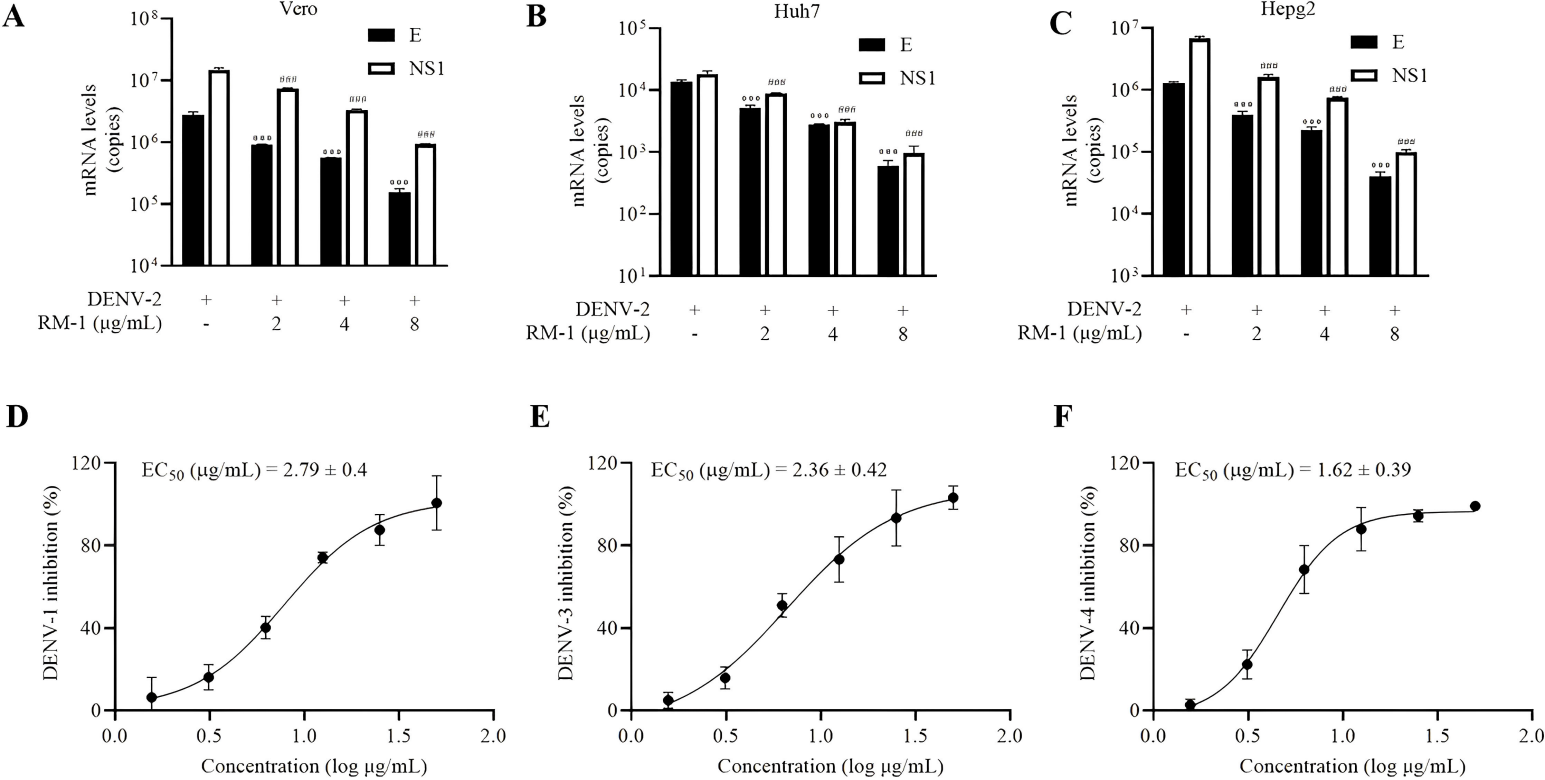
Serotype and cell type specificity of RM-1. The antiviral activities of RM-1 in Vero **(A)** Huh7 **(B)** and Hepg2 **(C)** cell lines measured by qRT-PCR. The EC_50_ of RM-1 in DENV-1 **(D)**, DENV-3 **(E)** and DENV-4 **(F)** monitored by CCK-8 assay. Data were expressed as mean ± SD of three independent experiments. ^###^ *P* < 0.001 *versus* DENV group in NS1 RNA, ^***^ *P* < 0.001 *versus* DENV group in the RNA level of E protein by one-way ANOVA with Tukey’s test.

To evaluate the antiviral specificity of RM-1 in different cells, a panel of different cell lines from other species origins, including Vero, Huh7 and HepG2, were used in this experiment. Interestingly, dose-dependent anti-DENV activities of RM-1 in all these cell lines were observed (shown in **Figures 4A–C**). These results provide definitive evidence that RM-1 is broadly active against DENV in multiple cell lines, suggesting its potential application as an extensive host-centered antiviral agent.

In order to assess the broad-spectrum activities of RM-1, another three serotypes of DENV (DENV-1, DENV-3 and DENV-4) were tested using the CCK-8 assay on BHK-21 cells. Similar findings were found that RM-1 showed antiviral activities in these virus strains. The EC50 values were found to be 2.79 μg/mL for DENV-1, 2.36 μg/mL for DENV-3, and 1.64 μg/mL for DENV-4, respectively (shown in **Figures 4D–F**). Based on these data, we suggest that RM-1 might serve as a promising broad-spectrum DENV inhibitor.

### 3.5 RM-1 improves clinical manifestations and survival rate in DENV-infected mice

**Figure 5:**
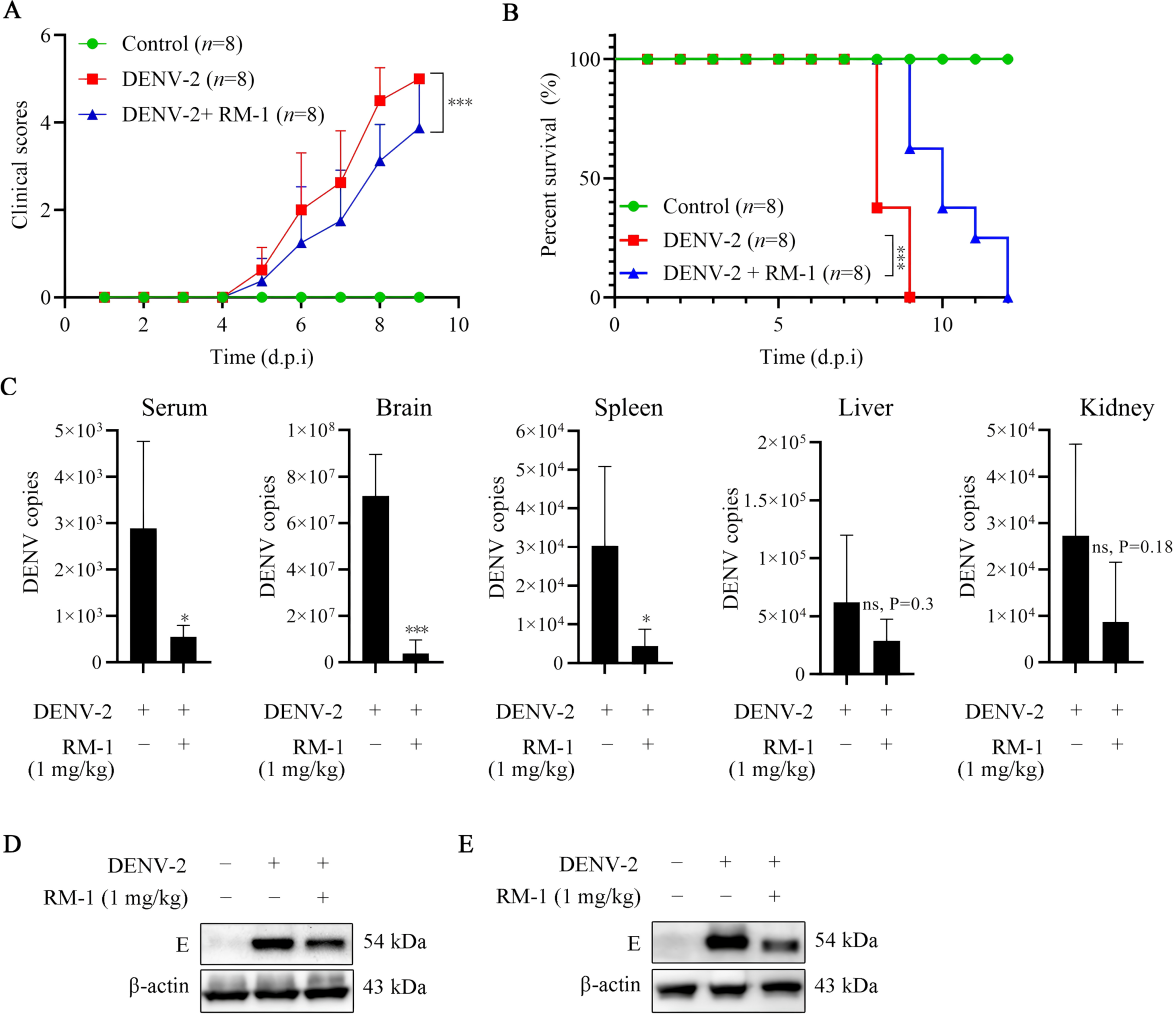
Effects of RM-1 on mortality and viral replication in DENV-infected suckling mice. 7-day-old ICR suckling mice were infected with DENV-2 by both intracerebral (400 PFU) and intraperitoneal (4×10^5^ PFU) injections in the absence or presence of RM-1 (1 mg/kg). The disease manifestations **(A)** and survival **(B)** of mice were monitored daily after virus infection. Disease manifestations were scored as follows: 0 for health, 1 for minor symptoms (reduced mobility and hunched posture), 2 for limbic seizure, 3 for moving difficulties (anterior limb or posterior limb weakness), 4 for paralysis and 5 for death. **(C)** The mRNA levels of E protein in serum, brain, liver, spleen and kidney were analyzed at 6 d.p.i. by qRT-PCR. The expression of E protein in brain **(D)** and spleen **(E)** were analyzed at 6 d.p.i. by Western blot assay. Data were expressed as mean ± SD of three independent experiments. *P < 0.05 versus DENV group, ***P < 0.001 versus DENV group, ns indicates no significant difference (P> 0.05) as determined by Student’s t test.

To further evaluate the antiviral effects of RM-1 in vivo, a DENV-infected suckling mouse model, previously reported, was established (18). RM-1 (1 mg/kg) was injected intraperitoneally at 0, 1, 3, 5 and 7 d.p.i. Notably, at 9 d.p.i., the DENV + RM-1 group exhibited a significant reduction in clinical scores compared to the DENV-infected mice group (shown in **Figure 5A**). The survival analysis revealed that DENV-infected mice experienced severe illness, resulting in death within 5 to 9 d.p.i, while RM-1 remarkably delayed the DENV-induced mortality by 3 d.p.i. (shown in **Figure 5B**). These results demonstrate that RM-1 protected mice against DENV-2-induced illness and lethality.

### 3.6 RM-1 reduces viral loads and ameliorated the pathological changes in DENV-infected mice

To study whether RM-1 could protect DENV-infected mice through regulating virus amplification, the viral loads in serum and various tissues were assessed using qRT-PCR. As illustrated in **Figure 5C**, high viral loads of serum, brain, spleen, liver and kidney induced by DENV-2 were observed. RM-1 considerably decreased the genomic viral copies in the serum, brain and spleen, while no notable differences were detected in other tissues. Moreover, the decreased expression levels of E protein detected by Western blot analysis further validated that RM-1 reduced the viral loads in brain (shown in **Figure 5D**) and spleen (shown in **Figure 5E**).

**Figure 6:**
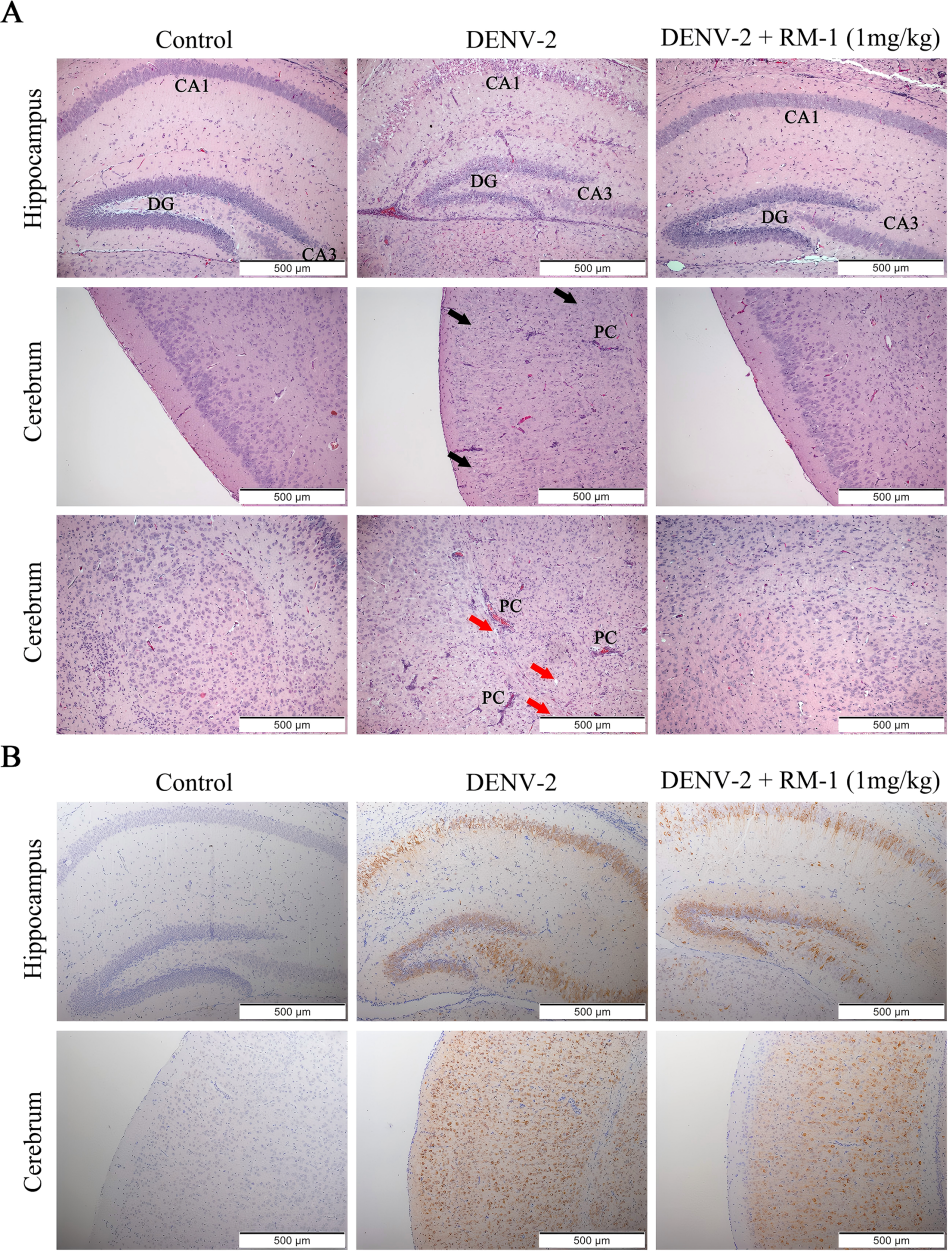
Effects of RM-1 on the pathological changes of brain in DENV-infected suckling mice. **(A)** Histopathological changes in the hippocampus and cerebrum were evaluated by hematoxylin & eosin staining. The black and red arrows indicated the loss of neurons in hippocampus (DG, dentate gyrus; CA1 and CA3 areas) and cerebrum (black arrows), respectively. The infiltration of immune cells, perivascular cuffs (PCs) and edema (red arrows) were also observed under an Olympus IX 53 light microscope (100 ×). **(B)** Immunohistochemical staining for anti-E protein in hippocampus and cerebrum.

To further investigate the effects of RM-1 on the pathological changes of brain and spleen, H&E staining was performed. We noted that the intense loss of pyramidal neurons in the hippocampus (DG, dentate gyrus; CA1 and CA3 regions) and the cerebrum, perivascular cuffs (PCs) of mononuclear cell infiltration and the edema in multifocal areas were widely distributed across the brains of DENV-infected mice at 6 d.p.i. However, all these brain tissue damages were significantly ameliorated by RM-1 (shown in **Figure 6A**). Unexpectedly, there were no pathological changes observed in spleen between DENV-infected mice and control group (data not shown). Besides, immunohistochemical staining revealed that positive cells for the E protein were primarily localized in the areas of inflammation and tissue damage in DENV-infected mice, while RM-1 significantly decreased the positive areas (shown in **Figure 6B**). Overall, these results show that RM-1 reduced viral replication in the brain and spleen and mitigated the pathological deterioration of the brain in DENV-infected mice.

## 4 Discussion

Andrographolide, a bicyclic diterpenoid lactone from Andrographis paniculata, exhibits definite therapeutic effects and has been extensively utilized in clinical settings for the treatment of various infectious diseases (19). Several researches have demonstrated that andrographolide shows anti-DENV effects with a EC_50_ of around 22 μM (20). In our study, we found RM-1, an andrographolide derivative, has stronger antiviral effects with a EC_50_ of around 2.24 μg/mL than andrographolide, suggesting the more antiviral potential of this derivative compared to andrographolide. To our knowledge, this is the first report highlighting the anti-DENV activities of RM-1.

DENV enters the host cells through three-stage process: receptor binding, endocytosis and fusion. Once inside the cell, viral RNA was released into the host cell cytoplasm where the viral replication process was initiated (21). We used a time-of-addition assay to explore at which stage RM-1 exerted its antiviral roles. Besides, in order to distinguish between viral attachment and entry/fusion, a temperature regulation between 4°C (allowing virus attachment but preventing entry) and 37°C (enabling virus entry and fusion) was allowed during the co-incubation of virus and cells (10). Our results demonstrated that the inhibition of viral infection was appeared only in the attachment stage. Furthermore, when cell monolayers were pretreated with RM-1 for 5 h, no improved cell survival was observed compared with DENV-infected cells, revealing that the antiviral activities of RM-1 do not owe to targeting to cellular receptors. Therefore, we infer that RM-1 could target the sites of DENV directly, which may have the potential to serve as a direct-acting antiviral (DAA).

In recent years, DAAs have revolutionized the viral treatment. Quite a number of DAAs have been widely serviced in clinical to treat HCV (22), HIV (23), influenza viruses (24) and so on. The broad spectrum of antiviral activities is crucial for the development of DAAs. The results across different cell lines showed that the antiviral effects of RM-1 were independent of cell type. In addition, different serotypes of DENV affect the sensitivity of the DAAs (25). Previous studies have reported that the anti-DENV activities of andrographolide only occurred in DENV-2 and DENV-4 serotypes. In this study, we clearly found that RM-1 inhibited the replication of all four DENV serotypes, indicating its broad-spectrum antiviral effects. The slight differences in antiviral activities among these strains can be attributed to conformational changes in the target protein, which may alter the binding affinity or accessibility of the ligand, thus influencing the overall antiviral efficacy (26).

After clarifying the anti-DENV activities of RM-1 *in vitro*, we observed that RM-1 alleviated the clinical symptoms and extended the survival of DENV-infected suckling mice. Except survival rate, encephalitis-like symptoms, and cerebral lesions, previous DENV infection models in suckling mice have generally overlooked other systemic damage caused by the virus (27, 28). In our study, viral nucleic acids in DENV-infected sucking mice were detected in serum, spleen, liver and kidney as well as the brain, which were consistent with the detection results in AG129 mice models infected with DENV (29). Interestingly, in addition to the serum and brain, the viral loads in spleen were dramatically reduced by the intraperitoneal injection of RM-1, while no significant changes in liver and kidney. This may be due to the fact that RM-1 stimulates the interferon-gamma (IFN-γ) secretion of T lymphocytes in spleen, similar to andrographolide (30, 31). Nonetheless, pathological damages among these tissues were only occurred in the brain of DENV-infected suckling mice, likely because other organ lesions had not yet manifested before the onset of mortality. Although the antiviral activities of RM-1 have been well identified in suckling mice, further confirmation of these effects in a DENV-infected adult mice model, such as AG129 mice, is needed.

The E protein initiates contact with the potential cell receptors and mediates the subsequent endocytosis process (32). Drugs targeting E protein have already been successfully applied in clinical practice, such as Arbidol, an anti-influenza drug (26). DIII (an immunoglobulin-like β-barrel structure) of E protein exposes on DENV surface and is characterized by the receptors binding sites of E protein (33). Several pieces of evidence indicate that targeting ED III inhibits the replication of DENV. For example, antibodies targeting ED III effectively block DENV attachment to Vero cells (34). Additionally, recombinant ED III has been found to inhibit DENV entry into mammalian cell lines such as BHK-21 and HepG2, as well as the mosquito cell line C6/36 (35, 36). Furthermore, several small molecules and peptides have been identified as possessing anti-DENV properties by targeting ED III (37–40). Although the RM-1-ED III interaction was further demonstrated by SPR (shown in **Supplementary Figure S1**), the possibility of such interaction in cells or tissues is still not revealed. In addition, whether RM-1 targets these amino acid residues needs further experimental proof.

To summarize, RM-1, an andrographolide derivative, was firstly found to exert antiviral activities against DENV in the lower micromolar range compared to andrographolide. Our studies not only adequately identified this effect both *in vitro* and *in vivo*, but also suggested that the underlying mechanism might be related to blocking viral attachment process through targeting the DENV ED III. These results support that RM-1 may become a promising antiviral candidate of attachment inhibitors for the treatment of dengue fever in the future.

## 5 Conclusion

In this study, we demonstrated that RM-1 exerts potent antiviral activity against dengue virus (DENV) by blocking viral attachment to the cell surface. *In vitro* experiments showed that RM-1 specifically inhibited DENV-2 attachment with an EC_50_ of 2.24 μg/mL, accompanied by a significant reduction in cytopathic effects (CPE) in a dose-dependent manner. Mechanistically, RM-1 suppressed the synthesis of progeny DENV-2, as evidenced by decreased viral plaque formation, and downregulated the RNA and protein expression of viral structural protein E and non-structural protein NS1. Notably, RM-1 exhibited broad-spectrum antiviral activity against all four DENV serotypes (DENV-1 to -4) and was effective in multiple cell lines (BHK-21, Vero, Huh7, HepG2), suggesting its potential as a host-centered antiviral agent.

*In vivo* studies in DENV-infected suckling mice further confirmed that RM-1 (1 mg/kg) improved clinical scores, prolonged survival, and reduced viral loads in serum, brain, and spleen. Histopathological analysis revealed that RM-1 ameliorated DENV-induced brain damage, including neuronal loss, perivascular inflammation, and edema, while decreasing viral E protein expression in affected tissues. Collectively, these findings highlight RM-1 as a promising candidate for developing broad-spectrum antiviral therapeutics against DENV infections, with its mechanism of action targeting early viral attachment and subsequent replication.

## Data Availability

The original contributions presented in the study are included in the article/**Supplementary Material**. Further inquiries can be directed to the corresponding authors.
