# Supplementary material for: Identification of Rhododendron mariae extraction as a new attachment inhibitor against dengue virus by targeting the envelope protein domain III
Source: Front Immunol. 2025 Aug 13;16:1663878. doi: 10.3389/fimmu.2025.1663878 (PMC12380535; doi:10.3389/fimmu.2025.1663878)
Supplement: Supplementary file 1 [file DataSheet1.docx]

**Supplemental Material**

Table S1. Key reagents and antibodies in this article.

| Reagent/Antibody | Source | Identifier |
| --- | --- | --- |
| Dengue virus NS1 protein antibody | GeneTex | Cat# GTX124280  RRID: AB_11171351 |
| Dengue virus Envelope(E) protein antibody | GeneTex | Cat# GTX127277  RRID: AB_11163414 |
| Anti-rabbit IgG, HRP-linked Antibody | Cell Signaling Technology | Cat# 7074S  RRID: AB_2099233 |
| Alexa Fluor 488-conjugated anti-Rabbit IgG antibody | Invitrogen | Cat# A31566  RRID: AB_10374301 |
| 4′, 6-diamidino-2-phenylindole (DAPI) | Bioss | Cat# S0001 |
| BCA protein assay kit | Thermo Fisher Scientific | Cat# 23227 |
| Enhanced chemiluminescence (ECL) kit | Merck Millipore | Cat# WBULS0500 |

**RM-1 inhibits DENV infection by interacting with ED III**

To experimentally verify whether RM-1 could interact with ED III, SPR assay was implemented. RM-1 exhibited dose-dependent binding to ED III, with a dissociation constant (KD) value of 707 nM, which reveals the tight bond between RM-1 and ED III (Fig. S1D). Overall, these findings suggest that RM-1 targets ED III of DENV to exert its antiviral effects.

**Fig. S1**

**
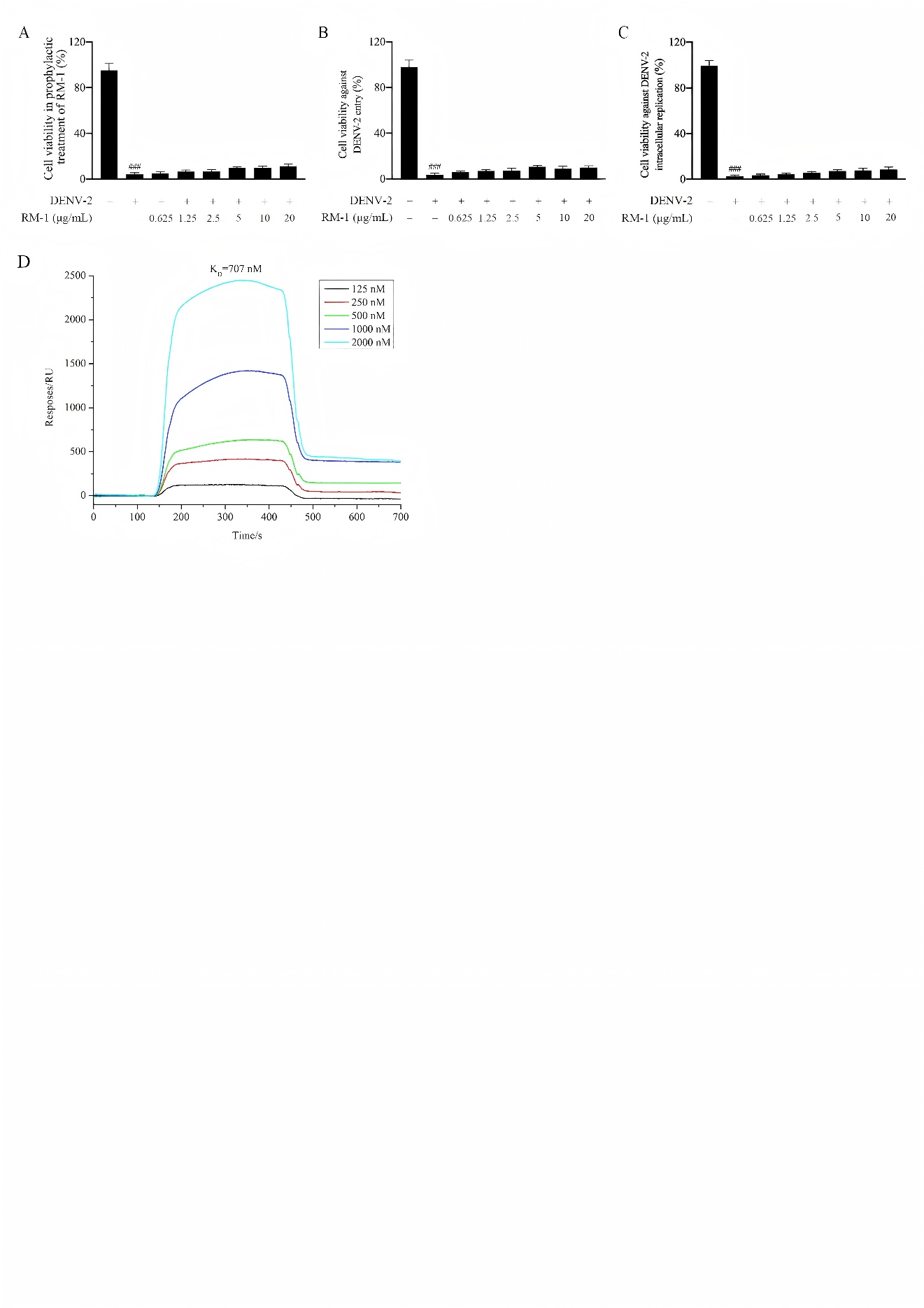
**
